# Patient Radiation Exposure during Enteroscopy-Assisted Endoscopic Retrograde Cholangiopancreatography in Surgically Altered Anatomy (with Video)

**DOI:** 10.3390/diagnostics14020142

**Published:** 2024-01-08

**Authors:** Laurent Monino, Tom G. Moreels

**Affiliations:** Department of Gastroenterology & Hepatology, Cliniques Universitaires Saint-Luc, 1200 Brussels, Belgium; monino.laurent@hotmail.fr

**Keywords:** ERCP, enteroscopy-assisted-ERCP, surgically altered anatomy, fluoroscopy, radiation exposure

## Abstract

Background: Fluoroscopy must be used cautiously during endoscopic retrograde cholangiopancreatography (ERCP). Radiation exposure data in patients with surgically altered anatomy undergoing enteroscopy-assisted ERCP (EA-ERCP) are scarce. Methods: 34 consecutive EA-ERCP procedures were compared with 68 conventional ERCP (C-ERCP) procedures. Patient and procedure characteristics and radiation data were collected. Results: Surgical reconstructions were gastrojejunostomy, Roux-en-Y hepaticojejunostomy, Roux-en-Y total gastrectomy, Roux-en-Y gastric bypass and Whipple’s duodenopancreatectomy. Procedures were restricted to biliary indications. Mean fluoroscopy time was comparable in both groups (370 ± 30 s EA-ERCP vs. 393 ± 40 s C-ERCP, *p* = 0.7074), whereas total mean radiation dose was lower in EA-ERCP (83 ± 6 mGy) compared to C-ERCP (110 ± 11 mGy, *p* = 0.0491) and dose area product (DAP) was higher in EA-ERCP (2216 ± 173 µGy*m^2^) compared to C-ERCP (1600 ± 117 µGy*m^2^, *p* = 0.0038), as was total procedure time (77 ± 5 min vs. 39 ± 3 min, *p* < 0.0001). Enteroscope insertion to reach the bile duct during EA-ERCP took 28 ± 4 min, ranging from 4 to 90 min. These results indicate that C-ERCP procedures are generally more complex, needing magnified fluoroscopy, whereas EA-ERCP procedures take more time for enteroscope insertion under wide field fluoroscopic guidance (increased DAP) with less complex ERCP manipulation (lower total radiation dose). Conclusions: Radiation exposure during EA-ERCP in surgically altered anatomy is different as compared to C-ERCP. EA-ERCP takes longer with a higher DAP because of the enteroscope insertion, but with lower total radiation dose because these ERCP procedures are usually less complex.

## 1. Introduction

Endoscopic retrograde cholangiopancreatography (ERCP) combines both endoscopy and fluoroscopy to visualize the biliary tree and the pancreatic duct in order to perform therapeutic endoscopic interventions. The technique was first described in 1968 and evolved rapidly and steadily since then [1]. It is nowadays considered an advanced therapeutic endoscopic procedure to treat both biliary and pancreatic pathological conditions. However, there is a considerable risk of ERCP-related adverse events, the most important ones being post-ERCP pancreatitis and cholangitis, post-sphincterotomy bleeding and perforation [2]. Therefore, ERCP requires rigorous and continuous training [3]. Apart from the risks related to the endoscopic intervention, the use of fluoroscopy during the procedure leads to potentially hazardous radiation exposure of both the patient and the medical staff involved in the procedure. The health-related risks of radiation exposure in the clinical setting are well-known and, therefore, specific safety precautions are to be taken when performing ERCP [4]. Education and training is mandatory for all personnel involved in the medical use of X-rays. In order to provide standardized information, major international endoscopy societies have published guidelines on the measures to be taken for radiation protection in digestive endoscopy [5,6]. Personal radiation protection measures for staff include the correct working position around the patient in relation to the X-ray source, the use of radiation protection shields, radiation protection aprons, collars and glasses, and radiation dose monitoring with personal dosimeters. To limit radiation exposure of the patient undergoing ERCP, the ALARA (as low as reasonably achievable) principle should be adhered to by reducing the duration of fluoroscopic examination and the number of radiographs taken [7]. General ALARA measures include the positioning of the patient as far as possible from the X-ray source, which should be underneath the examination table, restriction of the fluoroscopy time with pulsed (instead of continuous) fluoroscopy at a low pulse rate and ‘last image hold’ instead of taking radiographs, minimizing the use of magnification and maximizing the use of collimation [5,6]. These principles will help to reduce patient exposure to possibly harmful radiation. After each endoscopic procedure using fluoroscopy, patient radiation dose must be estimated and recorded in the medical file. The Kerma area product (KAP), also known as the dose area product (DAP) is the most often used metric for patient radiation exposure [5,6,8]. It is expressed as the radiation dose in Gray (Gy) multiplied by the irradiated body surface: Gy*cm^2^ or µGy*m^2^ with 1 Gy*cm^2^ = 100 µGy*m^2^ [5,6,8]. Other radiation exposure parameters include fluoroscopy time, total radiation dose, radiation dose per minute and number of single radiographs taken.

Several studies on patient radiation exposure during ERCP are available in the literature and DAP values of 20–50 Gy*cm^2^ are accepted as the dose reference level (DRL) for conventional ERCP in patients with normal anatomy by the European Society of Gastrointestinal Endoscopy [5,9]. However, little is known about patient radiation exposure during ERCP in patients with surgically altered anatomy of the upper gastrointestinal tract. Surgical interventions based on Billroth II variants with an afferent and an efferent limb and interventions based on Roux-en-Y variants with an alimentary, a biliopancreatic and a common limb complicate the endoscopic access to the biliary and the pancreatic system. These procedures often require the use of longer endoscopes like pediatric colonoscopes or even device-assisted enteroscopes [10]. It was shown that device-assisted enteroscopy, using either single-balloon, double-balloon or spiral enteroscopy, allows one to perform ERCP in patients with different types of surgically altered anatomy when the biliopancreatic system is inaccessible with conventional duodenoscopes [10]. However, insertion of the enteroscope and identification and intubation of the afferent or biliopancreatic limb rely on fluoroscopic guidance, leading to additional patient radiation exposure during enteroscope insertion before starting the actual ERCP procedure in patients with surgically altered anatomy [11]. Fluoroscopy not only serves to identify the correct intestinal limb, which cannot always be identified based on the endoscopic aspect only, it also helps to estimate the remaining distance to the papilla or the hepaticojejunostomy [12]. In addition, performing ERCP using a device-assisted enteroscope is different when compared to conventional ERCP using a duodenoscope, not only with regard to the introduction and the position of the endoscope, but also with regard to the accessory catheters and stents [10]. The enteroscope is a forward-viewing endoscope without additional elevator, and the working channel is much longer, with a smaller diameter requiring dedicated accessory materials [13]. In patients with surgically altered anatomy, the biliopancreatic system is approached from within the jejunum (distal or retrograde approach), whereas in normal anatomy the papilla is accessed via the antegrade approach through the stomach. The different approach changes the orientation of the bile duct or the pancreatic duct during the ERCP procedure [13]. These technical aspects need to be taken into account when planning to perform ERCP in patients with surgically altered anatomy. Due to these difficulties, technical success rates of enteroscopy-assisted ERCP (EA-ERCP) are generally lower than those of conventional ERCP (C-ERCP) [14].

In order to estimate patient radiation exposure during EA-ERCP, we retrospectively compared metrics for radiation exposure from consecutive C-ERCP procedures in patients with normal anatomy with EA-ERCP procedures in patients with surgically altered anatomy during a 4 month period.

## 2. Materials and Methods

The 2 endoscopists involved in the current study are trained in conventional ERCP, device-assisted enteroscopy and enteroscopy-assisted ERCP. The endoscopy unit is a third-line referral center for these kinds of advanced endoscopic procedures. All C-ERCP and EA-ERCP procedures with a biliary indication performed by the 2 endoscopists during a 4 month period were retrospectively reviewed from the electronic medical patient files (Epic, Epic Systems Corp., Verona, WI, USA) and metrics for patient radiation exposure were compared: duration of the procedure (in min), time to reach the papilla or the hepaticojejunostomy in patients with surgically altered anatomy (in min), duration of the fluoroscopy time (in sec), total radiation dose entering the patient or air Kerma (in mGy), radiation dose per min (in Gy/min) and air Kerma product or dose area product (DAP) (in µGy*m^2^). ERCP procedures with a pancreatic indication were excluded from the analysis to allow a more homogenous group of study patients. Clinical patient data were also recorded: age and sex, type of surgical reconstruction, biliary ERCP indications and interventions. All consecutive patients were included in the analysis and radiation exposure metrics reflect daily clinical practice due to their retrospective analysis. All procedures were performed on a Siemens Luminos Agile Max fluoroscopy machine (Siemens Healthcare GmbH, Erlangen, Germany) introduced in 2016 with an annual provider’s maintenance service. The fluoroscopy machine was set up and controlled by the 2 endoscopists, who are certified for the use of fluoroscopy (national certificate of radiation protection with continuous annual education).

After an overnight fast, ERCP procedures were performed under general anesthesia with endotracheal intubation, with the patient in the prone position for C-ERCP procedures and in the supine position for EA-ERCP procedures. The prone position during C-ERCP using a side-viewing duodenoscope facilitates biliary cannulation and is considered more ergonomically aligned for the endoscopist [15]. The supine position during EA-ERCP allows better manipulation of the device-assisted enteroscope and its overtube, as well as external manual compression of the abdomen whenever needed during enteroscope insertion. Both the prone and the supine position in patients under general anesthesia with endotracheal intubation facilitate fluoroscopy and anatomical orientation of the biliopancreatic system as compared to the left lateral positioning of the patient. Prophylactic antibiotics were given when clinically indicated to prevent post-ERCP cholangitis, as was intrarectal indomethacine 100 mg administration to prevent post-ERCP pancreatitis [16]. All C-ERCP procedures were performed with the Olympus TJF-Q180V duodenoscope (Olympus Corporation, Tokyo, Japan). EA-ERCP procedures were performed with the Olympus single-balloon enteroscopes SIF-Q180 and XSIF-180JY (Olympus Corporation, Tokyo, Japan). The technical characteristics of these single-balloon enteroscopes were described previously [17]. These are 200 cm long enteroscopes with a balloon-loaded overtube and a 3.2 mm working channel. All endoscopic procedures in our unit are performed using CO_2_-insufflation to open up the gastrointestinal lumen. Fluoroscopy was used to perform the actual ERCP procedure, but it also served to guide the insertion of the single-balloon enteroscope into the correct intestinal limb to reach Vater’s papilla or the hepaticojejunostomy.

Statistical analysis (Excel Microsoft 365 for Windows, Microsoft, Redmond, WA, USA) between the two groups was performed using Student’s *t* test for continuous variables and Chi-square test to compare ratios. *p*-values of <0.05 were considered to be significantly different.

The study was approved by the local Ethical Committee (2021/07AVR/162) and all patients signed informed consent for the endoscopic procedure.

## 3. Results

A total of 102 consecutive biliary ERCP procedures in 66 patients were performed by the two endoscopists during the 4 month period. They represent 68 C-ERCP procedures and 34 EA-ERCP procedures. Since this was a series of consecutive ERCP procedures, with the exclusion of pancreatic indications, some patients underwent more than one ERCP procedure during the study period and not all patients needed a primary sphincterotomy of the intact papilla. The male–female ratio was comparable in both groups, whereas mean patient age was significantly older in the EA-ERCP group. ERCP indications and interventions, as well as surgically altered anatomy reconstructions, are shown in Table 1.

**Figure 1:**
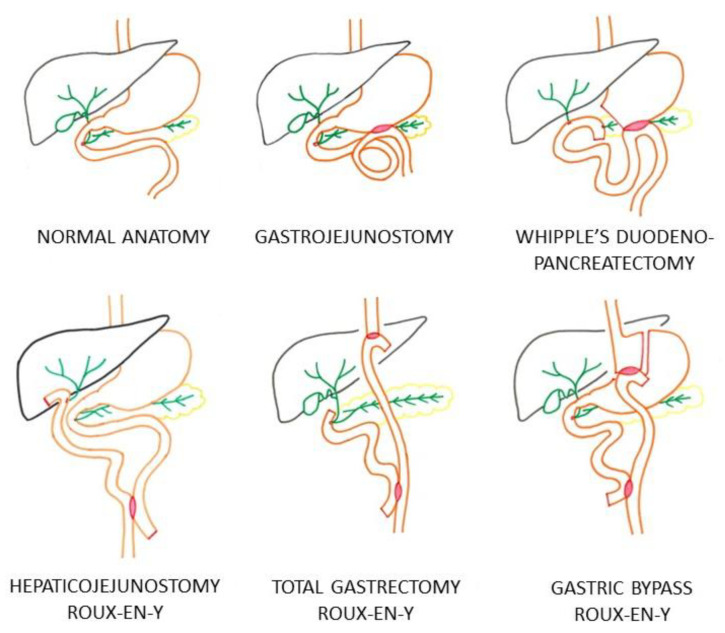
Different types of surgically altered anatomy in patients of group EA-ERCP. Surgical anastomoses are colored red.

Figure 1 illustrates the different types of surgically altered anatomy in the EA-ERCP group requiring a distal approach of the biliary system through either the intact papilla or through the hepaticojejunostomy. All C-ERCP procedures using a side-viewing duodenoscope were performed in patients with normal anatomy of the upper gastrointestinal tract. In the EA-ERCP group, all procedures were performed using a single-balloon enteroscope in patients with gastrojejunostomy in whom the papilla was reached through the gastrojejunal anastomosis, patients with Whipple’s duodenopancreatectomy with hepaticojejunostomy, and patients with Roux-en-Y reconstruction with either a hepaticojejunostomy or with an intact papilla (total gastrectomy and gastric bypass) (Table 1 and Figure 1).

In the EA-ERCP group benign anastomotic stricture at the level of the hepaticojejunostomy was the most frequent indication for biliary ERCP (44%), which was significantly higher than the ERCP indication for benign biliary anastomotic stricture after liver transplantation in the C-ERCP group (25%, *p* = 0.0498 Chi-square) (Table 1). Stricture at the level of the hepaticojejunostomy or at the level of the biliary anastomosis was often associated with the presence of common bile duct or intrahepatic biliary stones, resulting in balloon dilatation of the stricture, followed by stone extraction with or without additional plastic stenting. In contrast to the benign strictures, treatment of malignant biliary stricture was a frequent indication in the C-ERCP group (18%, *p* = 0.0091 Chi-square) and absent in the EA-ERCP group. Biliary plastic stenting was used more often in the C-ERCP group (54%) as compared to the EA-ERCP group (27%, *p* = 0.0075 Chi-square), whereas endoscopic balloon dilatation of benign strictures was used more often in the EA-ERCP group (61% vs. 31%, *p* = 0.0028 Chi-square). Moreover, in the EA-ERCP group, only 7 Fr stents were used, whereas in the C-ERCP group, both 7 Fr (30%) and 10 Fr plastic stents (70%) were used. Metallic stenting was not used very often in the current series of patients. There were no serious adverse events registered requiring redo of the endoscopic or surgical intervention related to the ERCP procedure in both groups.

**Figure 2:**
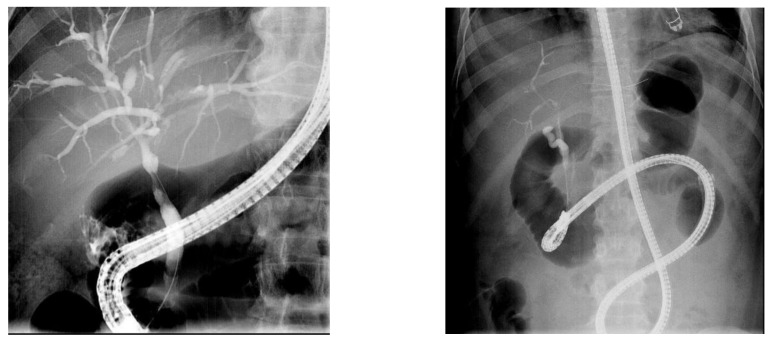
Fluoroscopic image of a C-ERCP in a patient with normal anatomy for a Klatskin tumor type I with high magnification: antegrade approach (**left image**). Fluoroscopic image of an EA-ERCP in a patient with Roux-en-Y gastric bypass-altered anatomy without collimation: retrograde approach (**right image**).

Patient radiation metrics of the C-ERCP group and the EA-ERCP group are shown in Table 2. The total procedure time (endoscope insertion + ERCP procedure) was significantly longer in the EA-ERCP group (77 ± 5 min) as compared to the C-ERCP group (39 ± 3 min, *p* = 0.0001 Student’s *t* test), which is mainly explained by the time needed to reach the papilla or the hepaticojejunostomy (28 ± 4 min, ranging from 4 to 90 min) during enteroscope insertion in the EA-ERCP group (Figure 2 and Video S1). The total radiation dose was significantly higher in the C-ERCP group (110 ± 11 mGy) as compared to the EA-ERCP group (83 ± 6 mGy, *p* = 0.0491 Student’s *t* test), indicating more complex C-ERCP procedures needing high magnification, whereas the DAP was significantly higher in the EA-ERCP group (2216 ± 173 µGy*m^2^ vs. 1600 ± 117 µGy*m^2^, *p* = 0.0038 Student’s *t* test). This radiation metric illustrates the use of wide-field fluoroscopy over the entire abdomen without collimation to guide the enteroscope towards and through the biliary or afferent limb during the phase of enteroscope insertion in patients with surgically altered anatomy (Figure 2).

## 4. Discussion

Potential health risks related to radiation exposure in medical practice are well known [4]. Since ERCP is an endoscopic procedure combined with fluoroscopy, specific radiation protection measures are mandatory, as outlined in international guidelines on radiation protection in digestive endoscopy [5,6]. To minimize harmful radiation exposure of the patient, protective measures according to the ALARA principle must be taken [7]. Whereas dose reference levels are available for ERCP procedures in patients with normal anatomy, little information is currently available on the ERCP-related patient radiation exposure in surgically altered anatomy. The current study investigated radiation exposure metrics of consecutive patients with different types of surgically altered anatomy undergoing EA-ERCP for biliary indications and compared these metrics with those of patients undergoing biliary C-ERCP in patients with normal anatomy by the same two endoscopists during the same study period. Both endoscopists are trained in C-ERCP and device-assisted enteroscopy, a proviso to start with EA-ERCP [13]. The European Society of Gastrointestinal Endoscopy (ESGE) suggests DAP dose reference levels of 20–50 Gy*m^2^ for conventional ERCP procedures, and comparable average DAP (13–66 Gy*m^2^) values were suggested in a recent review [5,9]. In the current study, the mean DAP for C-ERCP was 16.31 Gy*m^2^ (or 1631 ± 135 µGy*cm^2^) which is in accordance with these guidelines and with recent data from the literature, illustrating the correct use of fluoroscopy during the C-ERCP procedures in the current study [5,9,18,19]. The obtained DAP data are even at the lower end of the acceptable dose reference levels range. More recent literature data also show that these dose reference levels decrease with time over the years thanks to newer fluoroscopy machines on the one hand and the increased awareness of the risk of radiation exposure and the importance of radiation protection by the clinical staff on the other hand [20,21]. Stringent adherence to the ALARA principle may even reduce DAP dose reference levels for C-ERCP procedures to as low as 1–3 Gy*m^2^ [20,21]. Therefore, rigorous and continuous education in radiation safety is a mandatory aspect of ERCP training, focusing on parameters that minimize patient radiation exposure. The use of pulsed fluoroscopy, frame rate modification and collimation is strongly recommended to continuously reduce patient radiation exposure during ERCP procedures [22,23]. However, several studies have highlighted the lack of and the need for correct education in radiation protection during ERCP training [22,23].

The aforementioned DAP dose reference levels for ERCP procedures only take into account conventional ERCP using a duodenoscope in patients with normal anatomy [5]. In general, the introduction of the duodenoscope is straightforward under endoscopic guidance without the need for additional fluoroscopy [24]. Although EA-ERCP procedures are usually indicated for less complex benign biliary indications like postoperative strictures and bile duct stones, the total procedure time is longer. This is due to the often time-consuming and difficult phase of enteroscope insertion through the intestinal limbs of the surgically altered anatomy, ranging from 4 to 90 min in the current study with a mean of 28 ± 4 min. During this phase of enteroscope insertion, wide field fluoroscopy without collimation is used to identify the correct intestinal limb and to guide the device-assisted enteroscope through the limb and to assess the remaining distance to the papilla or the hepaticojejunostomy, as illustrated in the Video S1. The increased DAP in the EA-ERCP group (22.16 Gy*m^2^ or 2216 ± 173 µGy*cm^2^) is explained by this phase of enteroscope insertion with the use of fluoroscopy over the entire abdominal surface without collimation. This abdominal X-ray setting is not used during C-ERCP because fluoroscopic guidance is generally not needed to introduce the side-viewing duodenoscope through the normal upper gastrointestinal tract and to position it correctly at the level of the papilla in the second part of the duodenum. Although significantly higher than in the C-ERCP group, the DAP in the EA-ERCP group is still within the lower end of the acceptable range of dose reference levels suggested by the ESGE for conventional ERCP procedures [5]. However, based on recent studies, there is probably still room for improvement to further reduce radiation exposure thanks to more stringent adherence to the ALARA principle [20,21]. The results obtained in the current study may serve as a starting point to identify DAP dose reference levels in patients with surgically altered anatomy undergoing EA-ERCP using any type of device-assisted enteroscopy. It is currently not known, and probably of less clinical importance, whether DAP significantly varies with the type of surgically altered anatomy. The current study does not allow to differentiate and compare DAP values according to the type of surgically altered anatomy. This would require a higher number of ERCP procedures for each type of surgically altered anatomy. EA-ERCP DAP values should also be compared to radiation metrics of alternative approaches of biliary drainage like interventional endoscopic ultrasound or percutaneous transhepatic drainage or laparoscopy-assisted ERCP.

Complex ERCP procedures, like endoscopic treatment of malignant biliary obstruction or pancreatic indications, lead to increased radiation exposure through the use of high magnification fluoroscopic imaging in order to have a more detailed visualization of the biliopancreatic system [25]. This was also shown in the current study. There were more malignant biliary strictures to be treated in the C-ERCP group, requiring more stenting, resulting in a higher total radiation dose. In contrast, there were more benign biliary anastomotic strictures in the EA-ERCP group requiring balloon dilatation and less stenting. In this setting, we have adopted the following strategy in our unit when treating patients with surgically altered anatomy. Since endoscopic access to the biliary tree may be challenging, we prefer to treat benign biliary strictures by balloon dilatation first without stenting, as illustrated in Video S1 of this article. In cases of stricture recurrence and subsequent successful redo EA-ERCP, we opt for progressive stenting using plastic 7 Fr stents starting from the second EA-ERCP procedure. A third EA-ERCP procedure to remove or to replace stents will most likely be feasible after two previous successful procedures. There lies a risk in plastic stenting of the bile duct in patients with surgically altered anatomy. When the access to the biliary tree is very difficult due to the altered anatomy with long tortuous limbs and sharp intestinal angulations, it may be impossible to remove a previously placed biliary plastic stent during a second EA-ERCP attempt. Plastic biliary stents need to be removed or replaced every 3–4 months, and in case of failure, clothed plastic stents may induce obstructive cholangitis [26]. If a second redo EA-ERCP is successful, then progressive stenting may be considered safe without the risk of ending up with inaccessible plastic stents. Few patients with surgically altered anatomy undergo EA-ERCP for malignant biliary stricture, and in the current series there were none [14]. Malignant biliary strictures are usually treated using self-expandable metal stents (SEMS), which are often incompatible with the use of a long device-assisted enteroscope with a narrow working channel [27]. In the rare indication of malignant biliary strictures in patients with surgically altered anatomy, alternative more invasive approaches using endoscopic-ultrasound-guided, laparoscopy-assisted or percutaneous transhepatic access are available and may be even more effective [27,28]. Therefore, the preferred approach to the biliopancreatic system in patients with altered anatomy not only depends on the clinical indication but also on the type of surgical reconstruction and, above all, on the local availability and expertise of the different procedural techniques [29,30].

The current study provides dose reference levels for radiation exposure metrics like DAP in patients with surgically altered anatomy undergoing ERCP using device-assisted enteroscopy in daily clinical practice. However, there are also study-related limitations to be considered. First, the DAP values represent the outcomes of two well-trained endoscopists in a single center, with some patients undergoing more than one ERCP procedure. Second, not all patients presented with an intact papilla, which is usually more difficult to cannulate as compared to a post-sphincterotomy papilla or a hepaticojejunostomy. Third, due to the number of procedures in the current study, all EA-ERCP procedures were considered as one group, irrespective of the type of surgically altered anatomy. In general, long-limb altered anatomy with an intact papilla (like the Roux-en-Y gastric bypass) is considered more challenging as compared to a surgical reconstruction with normal length limbs and a hepaticojejunostomy without sphincter of Oddi when performing ERCP [13]. Future large-scale multicenter studies should be performed in order to confirm and consolidate or to adjust and define these dose reference values, both in patients with normal anatomy and in patients with different types of surgically altered anatomy.

## 5. Conclusions

The current study highlights differences in ERCP indications and the corresponding radiation exposure between conventional ERCP in patients with normal anatomy and enteroscopy-assisted ERCP in patients with surgically altered anatomy. Enteroscopy-assisted ERCP procedures take longer with a higher DAP (due to longer enteroscope insertion phase), but with a lower total radiation dose (thanks to less complex ERCP procedures). However, the DAP in EA-ERCP is still within the acceptable range of dose reference levels and can be used as a starting point to further reduce radiation exposure following the ALARA principle.

## Figures and Tables

**Table 1:** Patient and procedure characteristics.

	C-ERCP	EA-ERCP	*p*-Value
Patients (*n*)	43	23	
Procedures (*n*)	68	34	
Male/female ratio	24/19	16/7	0.2760
Age (y)	56 ± 4	67 ± 3	0.0343
Anatomy (*n* (%))			
Normal	68 (100)	-	
Gastrojejunostomy	-	3 (9)	
Hepaticojejunostomy Roux-en-Y	-	15 (44)	
Whipple’s duodenopancreatectomy	-	8 (23)	
Total gastrectomy Roux-en-Y	-	4 (12)	
Gastric bypass Roux-en-Y	-	4 (12)	
Indications (*n* (%))			
Anastomotic stricture	17 (25)	15 (44)	0.0498
Biliary stone(s)	20 (30)	9 (26)	0.7562
Anastomotic stricture + biliary stones	10 (15)	8 (24)	0.2705
Postoperative bile leak	3 (4)	2 (6)	0.7457
Malignant biliary stricture	12 (18)	0 (0)	0.0091
Indwelling biliary metal stent	3 (4)	0 (0)	0.2138
Benign biliary stricture after ampullectomy	3 (4)	0 (0)	0.2138
Endoscopic interventions (*n* (%)) multiple options			
Balloon dilatation	21 (31)	21 (61)	0.0028
Sphincterotomy	22 (32)	6 (18)	0.1167
Stone extraction	35 (52)	19 (56)	0.6739
Plastic stent placement	37 (54)	9 (27)	0.0075
Metal stent placement	5 (7)	1 (3)	0.3720
Stent removal	17 (25)	7 (21)	0.6205
Cholangioscopy (SpyScope)	3 (4)	0 (0)	0.2138

**Table 2:** Radiation metrics of C-ERCP and EA-ERCP.

	C-ERCP	EA-ERCP	*p*-Value
Total procedure duration (min)	39 ± 3	77 ± 5	0.0001
Fluoroscopy time (s)	393 ± 40	370 ± 30	0.7074
Total radiation dose (mGy)	110 ± 11	83 ± 6	0.0491
Radiation dose per min (mGy/min)	5.15 ± 0.59	4.25 ± 0.45	0.3059
Dose-area product DAP (µGy*m^2^)	1600 ± 117	2216 ± 173	0.0038

## Data Availability

The data presented in this study are available on request from the corresponding author. The data are not publicly available due to privacy reasons (patients can be identified from the data base).

## Supplementary Materials

The following supporting information can be downloaded at: https://www.mdpi.com/article/10.3390/diagnostics14020142/s1, Video S1: Enteroscopy-assisted ERCP in a patient with a Roux-en-Y hepaticojejunostomy following liver transplantation.
